# Precision Medicine in Hymenoptera Venom Allergy: Diagnostics, Biomarkers, and Therapy of Different Endotypes and Phenotypes

**DOI:** 10.3389/fimmu.2020.579409

**Published:** 2020-10-22

**Authors:** Simon Blank, Johannes Grosch, Markus Ollert, Maria Beatrice Bilò

**Affiliations:** ^1^Center of Allergy and Environment (ZAUM), Technical University of Munich, School of Medicine and Helmholtz Center Munich, German Research Center for Environmental Health, Member of the German Center of Lung Research (DZL), Munich, Germany; ^2^Department of Infection and Immunity, Luxembourg Institute of Health (LIH), Esch-sur-Alzette, Luxembourg; ^3^Department of Dermatology and Allergy Center, Odense Research Center for Anaphylaxis, University of Southern Denmark, Odense, Denmark; ^4^Department of Clinical and Molecular Sciences, Polytechnic University of Marche, Ancona, Italy; ^5^Allergy Unit, Department of Internal Medicine, University Hospital of Ancona, Ancona, Italy

**Keywords:** allergy diagnosis, biomarkers, immune tolerance, molecular allergology, precision medicine, venom allergen, venom-specific immunotherapy, Hymenoptera venom allergy

## Abstract

Allergic reactions to stings of Hymenoptera species may be severe and are potentially fatal deviations of the immunological response observed in healthy individuals. However, venom-specific immunotherapy (VIT) is an immunomodulatory approach able to cure venom allergy in the majority of affected patients. An appropriate therapeutic intervention and the efficacy of VIT not only depend on a conclusive diagnosis, but might also be influenced by the patient-specific manifestation of the disease. As with other diseases, it should be borne in mind that there are different endotypes and phenotypes of venom allergy, each of which require a patient-tailored disease management and treatment scheme. Reviewed here are different endotypes of sting reactions such as IgE-mediated allergy, asymptomatic sensitization or a simultaneous presence of venom allergy and mast cell disorders including particular considerations for diagnosis and therapy. Additionally, phenotypical manifestations of venom allergy, as e.g. differences in age of onset and disease severity, multiple sensitization or patients unsusceptible to therapy, are described. Moreover, biomarkers and diagnostic strategies that might reflect the immunological status of the patient and their value for therapeutic guidance are discussed. Taken together, the increasing knowledge of different disease manifestations in venom hypersensitivity and the growing availability of diagnostic tools open new options for the classification of venom allergy and, hence, for personalized medical approaches and precision medicine in Hymenoptera venom allergy.

## Introduction

Hymenoptera venom allergy (HVA) is one of the most serious IgE-mediated hypersensitivity reactions due to the high risk of severe and even fatal anaphylaxis. In the majority of patients, venom allergy can be effectively treated by venom-specific immunotherapy (VIT), the only available immunomodulatory and curative approach. However, a comprehensive diagnostic work-up, including the identification of the allergy-relevant venom, with different biomarkers and diagnostic tools is a prerequisite for proving clinically relevant sensitization and ensuring therapeutic success.

**Figure 1:**
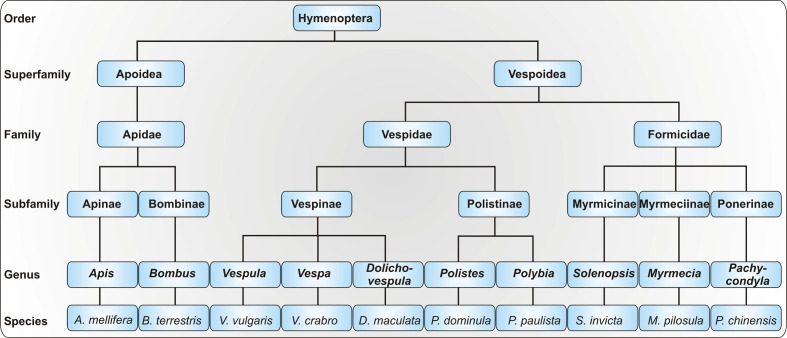
Taxonomy of allergy-relevant Hymenoptera. As the taxonomy of the order Hymenoptera is highly complex, only a selection of allergy-relevant taxa is shown. Displayed are exemplary species with particular relevance for Hymenoptera venom allergy.

HVA is caused by insects of the order Hymenoptera, which inject the venom as a defense mechanism. In Northern and Central Europe, the most common elicitors of venom allergy are honeybees (*Apis mellifera*) and yellow jackets (*Vespula* spp.). Venom allergy to hornets (*Vespa* spp.) is less common and it has been demonstrated that the vast majority of patients with anaphylactic reactions to hornet venom appear to be primarily sensitized to yellow jacket venom (YJV) (1). As bumblebees (*Bombus* spp.) are increasingly used for pollination in greenhouses, allergy to their venom has become more important but is still considered rare (2). In addition to honeybees and yellow jackets, paper wasp (*Polistes* spp.) venom allergy is of relevance in Southern Europe and Northern America. Allergy to the venom of other Polistinae such as *Polybia paulista* is prevalent in South America (3). Whereas allergies to stinging ants are rare in Europe, they are of great importance in Australia (jumper ant, *Myrmecia pilosula*), Asia (Asian needle ant, *Pachycondyla chinensis*) and America (fire ant, *Solenopsis invicta*). The taxonomy of allergy-relevant Hymenoptera is depicted in **Figure 1**.

The frequency of stings and, thus, of allergic reactions, depends on geographical, environmental, and ecological factors (4) which can rapidly change. For instance, *Polistes dominula*, domestic in Southern Europe, has invaded the United States (5), South Africa (6), and central Europe (7). Therefore, allergy to *Polistes dominula* venom (PDV) will probably become more important in the future. A second highly invasive Hymenoptera species, *Vespa velutina nigrithorax* (yellow-legged or Asian hornet), is gaining ground in Europe, although its natural habitat is tropical areas in Southeast Asia. Starting from France, it has spread rapidly across Europe, facilitated by suitable climatic conditions (8). *Vespa velutina nigrithorax* has become a common cause for Hymenoptera anaphylaxis in areas of Europe where it has become endemic (9).

In adults (> 18 years), 48.2% of cases of severe anaphylaxis are caused by insect stings (20.2% in children) (10). The prevalence of systemic sting reactions (SRs) in the adult population ranges between 0.3% and 8.9% and is lower in children (11). The estimated number of annual mortalities due to insect sting-induced anaphylaxis ranges from 0.03 to 0.45 per one million inhabitants (12). However, this number could be underestimated as many fatal reactions following insect stings may remain undetected (13). Large local reactions (LLRs) at the site of the sting that are characterized by a swelling with a diameter exceeding 10 cm and lasting for more than 24 h, occur in 2.4% to 26.4% of the general population (14).

**Figure 2:**
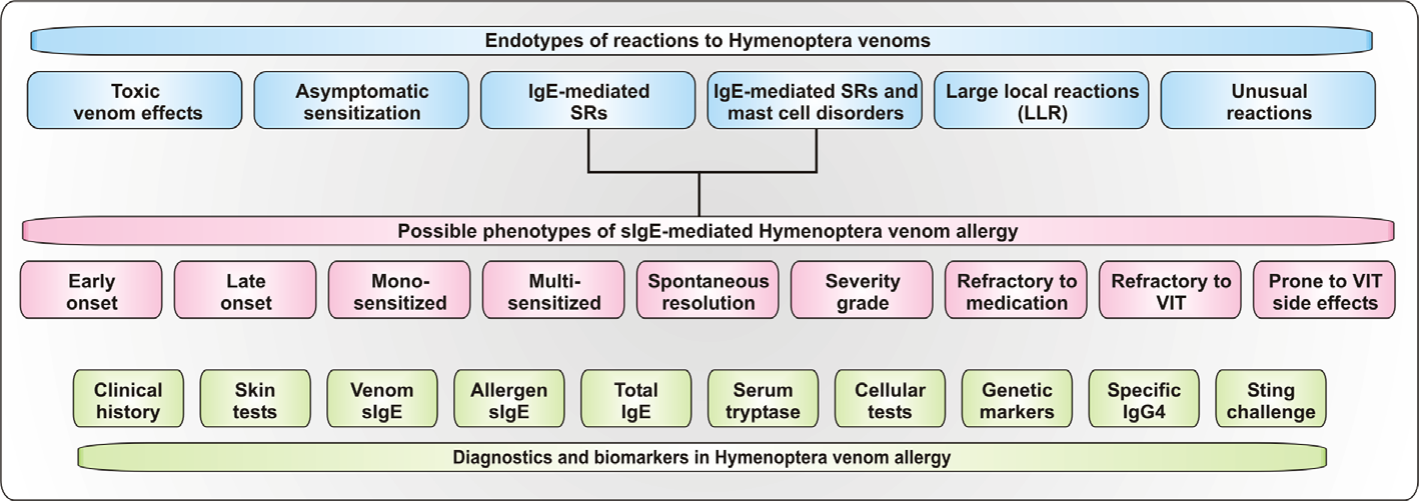
Endotypes und Phenotypes of reactions to Hymenoptera stings. Depicted are proposed endotypes of reactions to Hymenoptera venoms as well as of proposed phenotypes of IgE-mediated systemic allergic reactions. Additionally, available diagnostic tools and biomarkers for the assessment of the reaction are shown. SRs, systemic reactions.

The classification of allergic reactions to Hymenoptera venoms into different endotypes and phenotypes, which can be assigned through various biomarkers and diagnostic strategies (**Figure 2**), enables individual risk stratification for the patients and personalized therapeutic strategies.

## Endotypes and Clinical Manifestations of Hymenoptera Venom Allergy

Like other diseases, reactions to Hymenoptera stings can be divided into different endotypes, such as the physiological sting reaction in healthy individuals, IgE- and T cell-mediated allergic reactions, venom allergy in patients with mast cell disorders, asymptomatic sensitization and toxic or unusual reactions (**Figure 2**).

### Sting Reaction in Healthy Individuals / Toxic Venom Effects

Between 56.6% and 94.5% of the general population state to be stung by an insect of the order Hymenoptera at least once in their lifetime (12). The normal sting reaction consists of pain and inflammation (swelling, redness and itching) and is not dangerous. However, massive attacks with numerous sting events, for instance by Africanized honeybees, can be life-threatening to humans due to the toxicity of the venom (15–17). On rare occasions, single oropharyngeal stings can induce critical airway obstruction in non-allergic individuals by local swelling (18). If this reaction can be considered as LLR in the oropharynx is unknown so far.

### IgE-Mediated Systemic Reactions

In allergic individuals, already a single sting can lead to severe and fatal reactions (19). These IgE mediated reactions depend on an initial step of sensitization. During the encounter with a venom via stings, venom allergens enter the body. Antigen-presenting cells, such as dendritic cells, B cells or macrophages, incorporate, process and subsequently present the allergens to naive CD4^+^ T cells. These T cells either differentiate into Th1, Th2, or Th17 effector cells or take on a regulatory function as regulatory T cells (Treg). B cell class-switch and differentiation into IgE-producing plasma cells is induced by the cytokines IL-4 and IL-13 which are secreted by mature Th2 cells. After sensitization, the actual allergic reaction can occur if Fc epsilon receptors I (FcϵRI) on the surface of mast cells and basophils loaded with allergen-specific IgE antibodies are cross-linked during a further encounter with the allergens. This in turn leads to the degranulation of mast cells and basophils and the secretion of pro-inflammatory compounds such as histamine, proteases, cytokines, and lipid mediators. This mix of immunological active compounds leads to the induction of allergic symptoms in susceptible patients [for further information see Rindsjö and Scheynius 2010 (20)].

SRs can be mild (generalized skin symptoms such as urticaria or angioedema), moderate (e.g. dyspnea, gastrointestinal symptoms or dizziness) or severe (e.g. unconsciousness, anaphylactic shock, respiratory or cardiac arrest) (21). Of note, there is no necessary correlation between the severity of sting reactions at two different times (22). The worst-case scenario, anaphylaxis, is characterized by the involvement of at least two organ systems (23). The most frequently affected organs are the skin and mucosa, followed by the cardiovascular system. Gastrointestinal symptoms occur in one third of the patients (10). It has been reported that 0.7% to 2% of all cases of anaphylaxis are lethal (24) and anaphylactic deaths to insect stings occur in most cases within 15–20 min after exposure (25). SRs usually begin 10 to 30 min after the sting but can also arise faster (e.g. in individuals suffering from mast cell disorders) or slower (1–4 h), although being less life-threatening in the latter case (4). The severity of symptoms can be boosted by different risk and co-factors such as suffering from mast cell disorders, physical exertion, male sex or older age (26).

### IgE-Mediated Reactions in Patients With Concomitant Mast Cell Disorders

Mast cell disorders, such as mastocytosis, are common cofactors for severe allergic reactions to Hymenoptera venoms. Mastocytosis is a clonal, neoplastic and heterogeneous (cutaneous, systemic and rare subtypes) disorder characterized by proliferation and accumulation of mast cells in the skin, bone marrow and other tissues (27). Mastocytosis frequently involves the somatic c-kit D816V mutation and elevated baseline levels of serum tryptase (27). The prevalence of mastocytosis may be as high as 7.9% in patients suffering from HVA which is significantly higher than that of the general population (28). Similarly, HVA causes anaphylaxis in nearly 30% of patients with mastocytosis (29). In addition to higher incidence, there is also convincing evidence of a strong association of mast cell disorders with an increased severity of sting‐induced anaphylaxis (30). The anaphylactic reactions in patients with systemic mastocytosis are characterized in the majority of cases by the absence of angioedema and erythema and the predominance of cardiovascular symptoms, such as hypotension, leading to loss of consciousness (31). The Spanish Network on Mastocytosis (REMA) has built and validated a simple clinical score associated with both a high sensitivity and specificity to predict systemic mastocytosis among patients who present with mast cell activation symptoms in the absence of skin lesions (32). Of note, anaphylactic sting reactions in mastocytosis patients have previously been thought to also occur in the absence of specific IgE (sIgE) (33) due to potential pharmacological mechanisms of mast cell degranulation. However, with the introduction of new methods and parameters of evaluation in the diagnostic work-up, this historic diagnostic gap has been closed and sIgE can be detected in the vast majority of patients (34, 35). Importantly, negative sIgE and negative skin tests have been reported in up to 15% of patients with systemic mastocytosis and history of a systemic reaction to insect stings (36), thus, restricting them from VIT. A recent study demonstrated that in mastocytosis patients suffering from YJV-allergy diagnostic sensitivity can be improved by lowering the cut-off for positive sIgE detection without marked changes in specificity (34). Here, a cutoff of 0.17 kU_A_/l gave an acceptable sensitivity and specificity (83.6 and 85.0%, respectively). Indeed, sIgE levels between 0.1 and 0.35 kU^A^/l should be considered relevant in patients with a clear clinical history, irrespective of the presence of mast cell diseases (35, 37, 38). VIT may be less protective in patients with severe initial SRs and mastocytosis and/or elevated serum tryptase (>11.4 ng/ml). Therefore, for safety reasons, it should be prolonged in those patients; it remains unclear whether it should be given lifelong or after which duration of treatment it should be stopped (21).

### Asymptomatic Sensitization

Interestingly, the presence of sIgE does not necessarily imply clinically relevant venom allergy. Between 9% and 29% of the population are sensitized to Hymenoptera venoms without previous clinical history of a sting reaction (39, 40). For most of these patients it is likely that the sensitization is asymptomatic and, thus, of no clinical relevance (41). However, the possibility of a reaction to a future sting cannot be fully excluded. To date, no indications are available on how to effectively manage these cases (42).

### Large Local Reactions

LLRs are defined by edema, erythema and pruritus and supposed to be an IgE-dependent late-phase allergic reaction that follows the local recruitment and activation of Th2 cells, eosinophils, basophils and other leukocytes (43, 44). Most studies find positive skin tests for venoms or venom-sIgE in 70%–80% of patients with LLRs (45). It was demonstrated that only very few patients suffering from LLRs develop more severe reactions when re-stung by the same insect (46). However, a recent study showed that SRs occur more frequently after a previous LLR than reported by previous literature (47). Here, 24% out of 225 patients with a previous LLR developed a SR after the first field re-sting. Among the 35 patients clearly re‐stung by the same insect, according to their history, 11% reported a SR. A conclusive statement on the connection of LLRs and SRs is challenging.

### Unusual Reactions

In addition to the well described allergic reactions to Hymenoptera stings, a variety of extremely rare and unusual reactions may occur. Examples are serum sickness-like manifestations, thrombocytopenic purpura, hemolytic anemia, Schönlein-Henoch purpura, Guillain-Barré syndrome, vasculitis, glomerulonephritis and demyelinization-related neurological complications (48, 49). The pathogenesis of most of these unusual reactions remains unclear but might involve toxic, autoimmune and type II and III hypersensitivity reactions.

## Phenotypes in Hymenoptera Venom Allergy

Different phenotypes of IgE-mediated HVA can be described by the age of onset, the course and severity of the disease, sensitization profiles and the response to therapy (**Figure 2**).

### Age of Onset

Systemic insect sting reactions seem to be rare in children, ranging between 0.9%–3.4% for mild systemic and 0.5%–0.9% for severe SRs (50, 51). However, according to the European Anaphylaxis Registry, HVA is the second most frequent cause of severe reactions in children (20.2%) after food allergy (10). Most studies on the pediatric group reveal the predominance of skin symptoms (60% of cases) and dyspnea (52, 53) in the course of anaphylaxis in children as compared to adults where cardiovascular symptoms more frequently occur (39, 52, 53). Elderly patients develop severe SRs more often and the fatality rate is higher than in children and young adults (39). This might be due to the fact that the cardiovascular system in children is more efficient compared to adults, hence, even the possibility of self-limitation of anaphylactic reactions exists.

### Spontaneous Resolution of the Disease

Despite the high prevalence of asymptomatic sensitization (up to 29%), the prevalence of sting-induced SRs is low (41). Why some sensitized patients do not react to a future sting is still unknown, but it is probably due to loss of sensitization over time and, thus, spontaneous resolution (11). On the other hand, the risk for adults who experienced a first anaphylactic reaction to suffer from a SR to a further sting is not 100% but between 40% and 60%. In the remainder, symptoms may be less severe or even completely absent (54). The natural history of insect sting allergy differs between children and adults. Early studies found that children have a favorable prognosis regarding re-stings, both, in studies based on sting challenge (55) and field stings (56, 57). In particular, children with mild SR outgrow their HVA in the majority of cases (58, 59). However, in children not treated with VIT and who have a history of moderate to severe SRs, the risk of future SRs remains as high as 40% after 1 to 9 years, and as high as 30% in years 10–20 after anaphylaxis (58).

### Severity of the Disease

Allergic SRs may involve one or more organ systems (i.e. cutaneous, respiratory, gastrointestinal, neurologic and cardiovascular systems), while the simultaneous involvement of two or more organ systems during an acute allergic event is a prerequisite for the diagnosis of anaphylaxis (23, 60). Several classifications were proposed to assess the degree of severity of anaphylaxis, each of which has limitations (61–64). The reason why some sensitized subjects develop mild systemic symptoms while others experience severe, even fatal SRs is not completely understood, even though several risk factors are known. The combination of several concomitant factors, which include environmental, genetic and individual factors, may account for the occurrence of SRs in individual patients (11). Patient-related risk factors for severe SRs in the adult population are older age, clonal mast cell disorders and/or elevated basal serum tryptase and accompanying respiratory or heart diseases (30, 65, 66). Available data regarding potential effects of beta‐blockers and/or angiotensin-converting enzyme (ACE) inhibitors in coexisting venom allergy are inconclusive; further studies are required to assess the impact of specific cardiovascular comorbidities (30). Risk factors and co-factors for severe SRs after Hymenoptera stings in children were identified in atopy (asthma, allergic rhinitis, and atopic eczema) (50, 53, 67, 68) and exercise (69). Moreover, the severity of the reaction was also associated with the severity of asthma (67). However, these findings should be confirmed in larger pediatric populations. Taken together, the aforementioned data hints to the existence of several subgroups of phenotypes in relation to the severity of SRs.

### Mono- and Multi-Sensitization

Patients with a history of SRs might show positive test results with one, two, or multiple venoms in the following diagnostic work-up (42, 70, 71). Particularly when the allergy-eliciting insect could not be identified by the patient, these double or multiple sensitizations challenge decisions concerning the proper therapeutic strategy as they might be a result of true primary allergy to more than one venom, cross-reactivity between venoms or asymptomatic sensitization (42). Only in the first case is VIT with all relevant venoms recommended, while for other scenarios VIT with the primary sensitizing venom only is sufficient. Fortunately, diagnostic tools, which in many cases allow the differentiation between primary allergy and cross-reactivity, exist.

### Patients Refractory to VIT

Although VIT is an effective curative treatment in the majority of Hymenoptera venom-allergic patients, in some cases it is not able to induce immunologic tolerance. To date, the reasons for treatment failures during VIT remain unclear. Risk factors for VIT failure are HBV allergy, very severe sting reactions, SRs induced by VIT, clonal mast cell disorders and/or elevated baseline levels of serum tryptase and perhaps the use of ACE inhibitors (72). A recent retrospective multicenter study of HBV-allergic patients demonstrated that a dominant sensitization to Api m 10 (>50% of sIgE to HBV) is a relevant risk factor for treatment failure with an odds ratio of 8.44 (73). Furthermore, all patients who showed sIgE to Api m 10 that was higher than 60% of HBV sIgE were therapy non-responder. Nevertheless, in most cases in which standard VIT fails, increasing the dosage successfully induces tolerance (74). Risk factors associated with a loss of protection after discontinuation of VIT include those mentioned above and failure to achieve protection during VIT (72). As longer treatment periods are associated with a lower risk of relapse (75), prolonging treatment or even maintaining it lifelong can be a reasonable option to achieve or retain tolerance, especially for high-risk patients (21).

### Patients Refractory to Medication

Refractory anaphylaxis (unresponsive to treatment with at least two doses of minimum 300 μg adrenaline) is a rare form of a life-threatening hypersensitivity reaction with high mortality. Comprehensive data on its definition, prevalence and risk factors is missing. Using the data from the European Anaphylaxis Registry (11,596 cases in total), 42 cases of refractory anaphylaxis of different origin were identified and compared to a control group of severe anaphylaxis cases (n = 4820). Cases elicited by insects were very few (n = 8) and often due to bee stings (76). Specific risk factors were not identified in Hymenoptera venom-allergic patients. Rudders *et al*. reported that among 153 emergency department patients with systemic insect sting reactions who received adrenaline, 16% received a second dose, without evaluating their characteristics (77). Although studies have demonstrated an association between beta-blocker use (or multiple antihypertensive drugs) and increased anaphylaxis severity (regardless of the trigger), as evidenced by increased organ system involvement and hospital admission (78–80), it is not yet established whether taking beta-blockers influences the number of adrenaline doses needed, thus, identifying a particular phenotype unresponsive to adrenaline therapy in case of anaphylaxis is not possible, including venom anaphylaxis. A recent case control study in adults did not find a significant link between beta-blocker use and the need for increased adrenaline dosing among emergency department patients with anaphylaxis (81). This suggests that the effects of beta-blockers may not be as significant in the clinical routine as previously thought. The lack of response to initial adrenaline may be due to insufficient drug delivery secondary to reduced venous return (82). A very recent study advocated for rapid escalation with early intravenous fluid therapy in patients where anaphylaxis is refractory to initial intramuscular adrenaline, even in patients without obvious hemodynamic instability (83). Patients suffering from mast cell disorders and venom allergy may need more doses of adrenaline because of the increased severity of anaphylaxis (84, 85) due to massive mast cell activation. Therefore, they can be identified as a specific patient phenotype, also in regards to the refractoriness to pharmacological treatment.

### Patients Prone to Adverse Reactions During VIT

VIT may induce adverse reactions. In large multicenter studies, the frequency of SRs reactions during VIT ranges from 8% to 20% (86–88). A slightly elevated risk for SRs during VIT is observed in vespid venom-allergic patients with elevated baseline serum tryptase levels, while this association was not found for treatment with HBV (88). Nevertheless, the most important risk factor for systemic adverse events with VIT (3.1- to 6-fold increased risk) is treatment with HBV (88–90). Although only shown in small patient populations, Api m 4 sensitization might be a risk factor for SRs during the up-dosing phase of VIT with HBV (91, 92). In a prospective study it was demonstrated that patients who had sIgE to Api m 4 >0.98 kU_A_/L show higher rates of SRs during the VIT induction phase (91).

According to the recent guidelines of the European Academy of Allergy and Clinical Immunology (EAACI) (21), in the case of systemic adverse events during the build-up phase of VIT, in addition to initially reducing the dosage, premedication with H1 antihistamines should be established. In case of repeated systemic adverse events during up-dosing, pretreatment with Omalizumab may be recommended (21). Currently, case reports and a case series have documented the usefulness of Omalizumab for the pre-treatment of patients who experienced systemic reactions to VIT, including patients with systemic mastocytosis (93–99). Most of these patients were able to tolerate VIT after Omalizumab pre-treatment. However, treatment regimens varied greatly. In some cases a single or a few injections before initiation of VIT were used (94, 98), while in other cases Omalizumab therapy and VIT were combined for several months (93, 97) or pre-treatment before every maintenance dose was administered (96). This suggests that the optimal treatment schedule with Omalizumab depends on the individual response to VIT.

## Allergens of Hymenoptera Venoms

Hymenoptera venoms are complex mixtures of a variety of substances which mediate the toxic effects. These include numerous proteins that represent potential allergens. In recent years, biochemical and molecular biological methods have made a significant contribution to the identification and characterization of new allergens of Hymenoptera venoms, shifting the focus from the whole venom to individual allergenic molecules (100).

To date, honeybee venom (HBV) is the best characterized Hymenoptera venom. In the last years, proteomic approaches have contributed to the identification of a variety of potential new allergens, including those of very low abundance (101, 102). Moreover, recombinant production strategies together with detailed immunologic analyses have enabled the identification of five major allergens in HBV (103): Api m 1 (phospholipase A2), Api m 2 (hyaluronidase), Api m 3 (acid phosphatase), Api m 5 (dipeptidylpeptidase IV), and Api m 10 (icarapin) with sIgE sensitization rates in HBV-allergic patient populations in the range of 57%–97%, 47.9%–52.2%, 49.6%–50%, 58.3%–61.7%, and 61.8%–72.2%, respectively (73, 103–109). Less information concerning sensitization rates is available for other HBV allergens and most of them appear to be of minor importance, not excluding that they might be of particular relevance for some patients. Bumblebee venom closely resembles HBV and both exhibit extensive cross-reactivity (110).

Similarly, the venoms of Vespoidea species are mostly alike (110). Shared between almost all of them is the highly abundant major allergen of unknown function named antigen 5. Moreover, most of the Vepoidea venoms contain phospholipases A1 as prominent and relevant allergens. The sensitization rates of YJV- and PDV-allergic patients to the phospholipases A1 (Ves v 1 and Pol d 1) and antigens 5 (Ves v 5 and Pol d 5) are 33.3%–54% and 87% and 84.5%–100% and 69%–72%, respectively (105, 108, 111–116).

HBV and Vespoidea venoms contain homologous allergens that can lead to cross-reactivity between the venoms. For instance, in addition to HBV, YJV and PDV contain dipeptitylpeptidases IV (Ves v 3 and Pol d 3) as major allergen (117, 118). Furthermore, hyaluronidases were identified in different Vespoidea venoms. However, in contrast to HBV Api m 2, their relevance as allergens in YJV (Ves v 2.0101 and Ves v 2.0201) seems to be limited (119).

## Diagnostics and Biomarkers in Hymenoptera Venom Allergy

**Figure 3:**
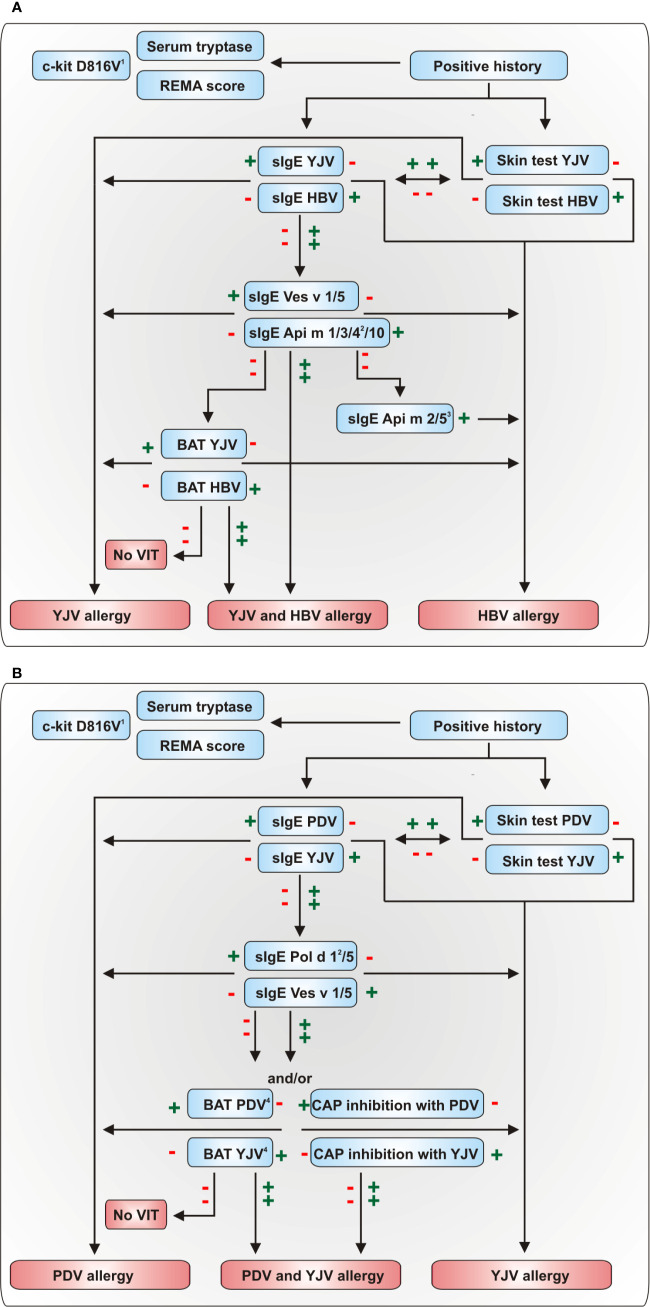
Diagnostic algorithms for the discrimination of **(A)** HBV and YJV allergy and **(B)** YJV and PDV allergy. The diagnostic algorithm presented in **(A)** can also be used to discriminate between HBV and PDV allergy using the *Polistes dominula* homologues of Ves v 1 and Ves v 5, Pol d 1 and Pol d 5, respectively. A plus indicates a positive and a minus a negative test result. ^1^In the majority of cases, positive PCR results proving the presence of the c-kit D816V mutation in peripheral blood mononuclear cells can confirm systemic mastocytosis (121). ^2^These allergens are only available for selected multiplex sIgE platforms. ^3^The HBV allergens Api m 2 and Api m 5 show potential cross-reactivity to not commercially available homologous allergens of YJV and PDV so that a positive test result does not necessarily preclude YJV or PDV allergy. ^4^BAT proved to be an effective tool for the assessment of double-positivity in HBV and YJV allergy (122). However, currently, no studies have analyzed its usefulness for the discrimination of PDV and YJV allergy. BAT, basophil activation test; HBV, honeybee venom; PDV, *Polistes dominula* venom; REMA score, score of the Spanish Network on Mastocytosis for predicting mast cell clonality and systemic mastocytosis in patients who experience anaphylaxis without cutaneous mastocytosis; VIT, venom-specific immunotherapy; YJV, yellow jacket venom.

Diagnosis of HVA comprises the clinical history of a systemic sting reaction and the proof of sensitization to the relevant venom by *in vivo* or *in vitro* testing (21, 39, 120). For successful VIT, the correct venom for treatment is of major importance. Due to the pronounced cross-reactivity between venoms, choosing the right venom for therapy is a challenging task if the patient was not able to identify the allergy-eliciting insect. Nevertheless, several advanced diagnostic tools and biomarkers exist (**Figure 2**) that facilitate accurate diagnosis and contribute to personalized risk stratification in HVA. Diagnostic algorithms to discriminate between HBV and vespid venom and YJV and PDV allergy are given in **Figures 3A, B**, respectively.

### Clinical History

The verification of a previous SR by clinical history should build the basis for a subsequent diagnostic work-up (**Figure 3**) since asymptomatic sensitization to Hymenoptera venoms is observed frequently (39, 40). A thorough clinical history includes information on number and date of sting reactions, symptoms, severity and time course of the reaction as well as the applied treatment. Additionally, individual risk factors for anaphylaxis such as mast cell disorders, medication, cardiovascular risks and other diseases as well as frequent exposition to relevant insects should be considered.

A special focus during the assessment of the patients’ history lies on the identification of the culprit insect. However, as many patients (and even allergy specialists) are not able to discriminate different Hymenoptera species (123, 124), all information has to be used with care and verification of the responsible insect with additional diagnostic tests is necessary.

### Skin Tests

Both, skin testing with venom extracts and sIgE measurements, should be performed in patients with a history of a SR (72). Skin testing with venom extracts can be done either as skin prick or intradermal testing following different protocols (125). Skin prick tests are performed at a concentration between 1 and 100 μg/ml, while an initial concentration in the range of 0.001–0.01 µg/ml is sufficient for intradermal tests. It should then be increased tenfold per step to a maximum concentration of 1 μg/ml (126). The sensitivity of skin prick test alone is around 64%, while the combination of prick test and intradermal test reaches a sensitivity of 94% (72). Despite a low risk of SRs (127), many institutions recommend to perform a graduated approach for skin testing (126). The simultaneous intradermal testing with different venoms is safe and efficient (127). Since the intradermal test is more sensitive, it should be used to confirm negative skin prick test results. Skin tests should be done at least 2 weeks after the sting reaction to avoid false-negative results during the refractory period (128). In case of negative tests despite a convincing history of a SR, skin tests should be repeated after 1–2 months. Of note, there is no correlation between the severity of a sting reaction and reactivity in skin testing (129).

### Baseline Serum Tryptase

It is recommended to determine the baseline tryptase level in the serum of all patients with a history of systemic sting reactions to identify patients at higher risk of developing severe reactions due to undiagnosed clonal mast cell disorders. High baseline levels in repeated measurements (particularly above 25 µg/ml) suggest mast cells disorders which need a further diagnostic work-up (e.g. by testing for somatic c-kit mutation or bone marrow analysis) (72). Adult patients with mast cell disorders and/or elevated baseline tryptase are not only at higher risk of more severe sting reactions but in some studies are also considered a risk population during VIT (28, 66, 130).

### Genetic Markers

Due to the increasing implementation of genome-wide association studies since the early 2000s, a multitude of different candidate genes with marker properties have been described. Most of these candidate genes have little or no clinical value and only a small fraction of the initial pool is being further investigated and implemented into the clinical routine. Nevertheless, genetic markers are an up-and-coming field in allergy research. One prominent example is the somatic c-kit D816V mutation which is used as minimally invasive secondary diagnostic criterion to confirm systemic mastocytosis, since >80% of patients with systemic mastocytosis are tested positive for this single nucleotide polymorphism (SNP) (131–133). As described earlier, systemic mastocytosis in combination with sensitization to Hymenoptera venom allergens is considered a risk factor for severe SRs. Therefore, c-kit D816V mutation is no direct genetic marker for venom allergy or increased risk of systemic allergic reactions but can offer added value to a thorough diagnosis and assessment of the individual risk.

A more straightforward marker is the polymorphism in the angiotensinogen AGT p.M235T gene which might be associated with more severe SR in patients with Hymenoptera venom allergy. Patients allergic to insect venoms have a higher prevalence of carrying this mutation and suffer more often, with an odds ratio of 2.5, from grade IV reactions (134).

Furthermore, a variety of studies focusing on the connection of HLA class I and class II genotypes and (venom) allergy have been published. For instance, HLA-B*18 and HLA-Cw*07 were significantly more frequent among Turkish bee- and/or wasp venom-allergic patients (135). Among HLA class II genotypes, DRB1*0101, DRB1*0103, DQA1*0101, and DQB1*0501 were found to be associated with an increased risk of being sensitized to Api m 1 (136). Still, to our knowledge, no conclusive statement regarding HLA class I and II frequencies and venom allergy or risk of SR is possible.

An elevated basal serum tryptase level might be caused by alfa-tryptasemia, a hereditary trait that was reported by Lyon et al. in 2014 (137). Affected persons carry additional copies of TPSAB1, the gene encoding for alpha-tryptase. Alpha-tryptasemia is discussed as one of the main sources for elevated serum tryptase and is associated with a 2–4 fold increased risk of systemic reactions (138, 139). The link between alpha-tryptasemia and mast cell activation disorder is part of ongoing research and not easy to assess (140).

### Total IgE

The measurement of the total IgE (tIgE) levels in combination with sIgE test results can be useful to improve and simplify interpretation. This is particularly important in connection with very low sIgE levels, since each sIgE level has a different relevance if produced in an environment with high or low tIgE values (141). Moreover, sIgE to Hymenoptera venoms is frequently observed in asymptomatic individuals with high tIgE (40, 142). Hence, the measurement of tIgE can provide guidance in the context of the ratio sIgE/tIgE, although it is not generally recommended in the guidelines.

### Specific IgE to Venom Extracts

Besides skin testing, the detection of sIgE to whole venom extracts is the most established diagnostic method to detect sensitization to Hymenoptera venoms. However, the diagnosis of clinically relevant allergy can only be made in combination with a corresponding clinical history. This also holds true for skin testing and other diagnostic approaches.

Although 0.35 kU_A_/L is commonly used as the lower threshold for sIgE detection, sIgE concentrations can be measured with high accuracy on the major singleplex sIgE immunoassay platforms with the lower end threshold of 0.1 kU_A_/L. Hence, sIgE levels between 0.1 and 0.35 kU_A_/L can be considered in the context of a clear clinical history (37, 143, 144). Ideally, sIgE measurements should be performed one to 6 weeks after the sting event. It should be kept in mind that negative sIgE test results in patients with convincing history of anaphylaxis can be caused by very low levels of sIgE or too long latency between the last sting and the diagnostic measurement (14, 35, 145).

Using the cut-off of 0.35 kU_A_/L, 90%–100% of HBV-allergic patients are tested positive for sIgE to HBV. The sensitivity of sIgE detection to YJV for YJV-allergic patients ranges between 83% and 97% (108, 116, 146). Nevertheless, sIgE testing of allergic patients with venom extracts in clinical routine frequently leads to multiple positive test results with different venoms. Intriguingly, for many of these patients only one venom is allergy-relevant. The clinical relevance of positive test results with other venoms with regard to systemic symptoms is limited (41, 147). However, as many patients are not able to identify the allergy-eliciting insect, clinically relevant allergy cannot be excluded. In addition to primary allergy to more than one venom, multiple positive test results with limited or no clinical relevance can be caused by: i) IgE antibodies directed to protein epitopes on homologous allergens present in the venoms, ii) sIgE to clinically irrelevant cross-reactive carbohydrate determinants (CCDs), and iii) asymptomatic sensitization (42). Hence, this often leads to unnecessary VIT with more than one venom, resulting in higher costs, potentially increased risk of side-effects and the possibility of *de novo* sensitization.

Overall, venom extract-based diagnostics has some pitfalls that complicate the differentiation of true primary allergy and cross-reactivity and, thus, the identification of the allergy-relevant venom and selection of the optimal therapeutic strategy.

IgE-inhibition tests with whole venom extracts can be used in particular cases to detect the primary sensitizing venom in patients double-positive to venoms without marker allergens, e.g. YJV and PDV (**Figure 3B**) (148–150). However, IgE-inhibition tests are costly, time-consuming and results occasionally difficult to interpret (149).

After an initial rise during the first months of treatment, sIgE levels to the respective venom decrease during VIT and usually remain low after discontinuation of VIT (151, 152). However, there is no evidence that they can be used as biomarker to predict success of therapy (21).

### Specific IgE to Individual Venom Allergens

In the recent past, the identification of relevant venom allergens has led to the development of molecular or component-resolved diagnosis (CRD) in HVA (42, 70, 153, 154). In CRD, sIgE against single allergens of venoms is determined. Thus, CRD not only provides information on whether a patient has sIgE against the whole venom, but also on exactly which allergens are relevant for a patient.

Due to the number of commercially available allergens, CRD has particularly increased diagnostic accuracy for the discrimination between HBV and YJV allergy. Diagnostic sensitivity of a combination of the two commercially available YJV allergens Ves v 1 and Ves v 5 ranges between 92% and 100% (35, 112, 113, 116, 155–158). CRD of HBV allergy is more complex in terms of diagnostic sensitivity. The first commercially available HBV allergen Api m 1 yielded a diagnostic sensitivity of 58% to 97% depending on the inclusion criteria of the patient population, geographical differences and sensitivity of the immunoassay platform used (103, 105–109). Hence, missing sensitization to Api m 1 does not exclude a genuine allergy to HBV. After the relevance of additional HBV allergens was demonstrated, these became available for routine diagnosis and it was shown that a combination of the allergens Api m 1, Api m 2, Api m 3, Api m 4, Api m 5, and Api m 10 leads to a diagnostic sensitivity of 94.4% in a population of HBV-allergic patients (103). However, this sensitivity might be lower in patients with sensitization to HBV only compared to those sensitized to both, HBV and YJV (159). Nevertheless, the extension of the panel of commercially available HBV allergens added clinical benefit as two-thirds of patients with negative sIgE to Api m 1 can be diagnosed using Api m 3 and Api m 10. In patients double-sensitized to HBV and YJV who were not able to identify the allergy-relevant insect, the combination of Api m 1, Api m 3, and Api m 10 increased the sensitivity of HBV allergy verification to 78.6% compared to 54% using Api m 1 only (104).

Recombinant allergens can be produced with the full protein epitope spectrum of the native allergens but without CCDs (160). Hence, positive sIgE test results indicate sensitization to protein epitopes only and not to CCDs, thereby excluding many clinically irrelevant sensitizations (106).

In addition to CCDs, cross-reactivity between different venoms can be caused by homologous allergens that share common IgE epitopes. The potential of CRD is evident from the fact that HBV and vespid venoms contain species-specific marker allergens (Api m 1, Api m 3, Api m 4, and Api m 10 for HBV and phopholipases A1 and antigens 5 for vespid venoms) in addition to homologous allergens. For many patients, the measurement of sIgE directed against these marker allergens allows the identification of the allergy-relevant venom and the discrimination between cross-reactivity and primary allergy (**Figure 3A**) (104). However, a clear limitation of the currently available CRD is the unavailability of potentially cross-reactive allergens of vespid venoms such as hyaluronidases and dipeptidylpeptidases IV as marketed allergens for sIgE detection, as they might be of relevance for particular patients.

While CRD is able to adequately distinguish allergies to HBV and vespid venom (particularly YJV), this is not the case when a differentiation between allergies to various vespid venoms is required. For instance, in Southern Europe, double-sensitization to YJV and PDV is much more frequent than to YJV/PDV and HBV (161–163). Although *Polistes* venom is devoid of CCDs (164), a definite discrimination is rarely possible due to the high degree of cross-reactivity between the major allergens of these venoms (110, 165). Moreover, only the PDV allergen Pol d 5 is available for CRD on the most common sIgE assay platform. Nevertheless, a previous study demonstrated that the measurement of relative levels of sIgE to the phospholipases A1 (Ves v 1 and Pol d 1) and antigens 5 (Ves v 5 and Pol d 5) of YJV and PDV allows the identification of the primary sensitizing venom in many cases (115). Therefore, the additional availability of these and other (e.g. dipeptidylpeptidases IV) cross-reactive allergens from vespid venoms for CRD would represent an added value for advanced precision diagnostics in HVA.

Additionally, some allergens may act as biomarkers for personalized risk stratification in patients undergoing VIT. As discussed in section *Patients Refractory to VIT*, dominant Api m 10 sensitization is a relevant risk factor for honeybee VIT (73). Thus, the knowledge of patient sensitization profiles allows choosing a therapeutic venom preparation for VIT that contains the highest amount of Api m 10 in a patient-tailored manner (73, 166, 167). Moreover, Api m 4 sensitization might be a marker to identify HBV-allergic patients with increased risk of SRs during the up-dosing phase of VIT (section *Patients Prone to Adverse Reactions During VIT*) (91, 92).

### sIgG4

With less than 5% of total IgG, IgG4 is the least abundant IgG subclass in human serum. However, IgG4 levels increase with chronic antigen exposure and are believed to induce immune tolerance and weaken inflammatory responses (168–171). IgE mediated hypersensitivity reactions are dampened by IgG4 by inhibiting IgE activity (172–174). Two different mechanisms have been proposed: i) IgG4 scavenges immunogenic epitopes on antigens and acts as a blocking antibody that prevents the downstream crosslinking of FcϵRI (175, 176). ii) IgG4 co-stimulates the inhibitory FcɣRIIb. This IgG receptor regulates signal transduction and inhibits the activation of effector cells (177).

VIT is associated with a significant increase in sIgG4 antibodies (178). However, after stopping VIT, sIgG4 levels start to decrease (179). In grass pollen allergy it was demonstrated that IgE-blocking capacity persisted for several years and correlated with clinical efficacy, although IgG4 levels rapidly decreased after stopping allergen-specific immunotherapy (172). This suggests that not the levels of sIgG4 but rather their functional activity might correlate with clinical efficacy and long term protection (180). Therefore, no evidence for the use of levels of venom-sIgG4 as biomarker for prediction of therapy success in VIT is given (21). Nevertheless, although IgG4 induction *per se* is no marker for therapeutic success, lack of IgG4 induction might be a marker for immunological unresponsiveness.

### Basophil Activation Test

The basophil activation test (BAT) mimics the activation of effector cells (basophils) responsible for IgE mediated allergic reactions *ex vivo*. Basophils in fresh patient blood are stimulated with allergens and the (up-)regulation of basophil specific markers, such as CD63 or CD203c, is observed.

Although BAT is not part of the routine diagnostics of venom allergy in all patients, it is well established and can be used in cases of unclear or negative skin and sIgE test results or when clinical history and diagnosis are contradictory. Studies demonstrated that BAT is able to detect sensitization in 81% of venom-allergic patients with negative sIgE and in 60% of patients that additionally exhibit negative intradermal skin tests (181, 182). Moreover, BAT is useful to correctly diagnose double-positive patients with inconclusive skin test or sIgE test results, particularly when the patient reacted only to one insect (122). Perhaps, the basophil response can also be used as biomarker for successful tolerance induction after VIT. It was demonstrated that, although unchanged after the first year of treatment, a significant and approximately fourfold decrease of basophil activation was observed in all tolerant subjects in response to submaximal allergen concentration after VIT (183).

BAT can further be used as biomarker to monitor ongoing VIT and to assess the success. Here, discrimination between BAT sensitivity and reactivity is needed. While the reactivity of basophils corresponds to the quantity of allergen needed to induce CD63 on the cell surface, the sensitivity is linked to the change of cell marker (e.g. CD63) amount (184). A successful VIT, which necessarily induces long term tolerance, decreases BAT sensitivity without changing the reactivity (183, 185, 186). Furthermore, a high sensitivity in BAT during the initial VIT phase is also associated with a higher risk of side-effects (186, 187).

### Sting Challenge Test

Due to the risk of severe reactions or *de novo* sensitization, sting challenge tests using living insects should not be used as diagnostic tool in untreated patients (188). However, apart from a well-documented field sting, the sting challenge test is the only recommended diagnostic method for the prediction of success of VIT (21). Moreover, a patient’s quality of life can be significantly improved by experiencing a tolerated sting challenge (189).

## Therapy of Hymenoptera Venom Allergy

Due to the high risk of very severe and even fatal reactions in venom-allergic patients, a careful patient management and proper therapeutic intervention is of major importance. Although some behavioral rules that might contribute to minimize the risk exist, avoiding stings completely is challenging. Therefore, patients with venom allergy should carry an emergency kit for self-administration including an adrenaline autoinjector as well as orally administered H1-antihistamine and corticosteroids. It is still a matter of debate, if the emergency kit should be carried during and after VIT as most patients are protected after reaching the maintenance dose (190).

VIT is the only disease-modifying and curative treatment of venom allergy that is able to efficiently protect patients against future severe sting reactions. VIT is recommended in adults and children with detectable sensitization and SRs exceeding generalized skin symptoms as well as in adults with generalized skin symptoms if quality of life is impaired (21). Although VIT is one of the most effective treatments in the field of clinical allergology, choosing the correct venom based on a comprehensive diagnostic work-up represents a crucial prerequisite for effective protection. Nevertheless, different biomarkers and diagnostic strategies are available that allow the classification into endotypes and phenotypes in HVA. Hence, they facilitate the correct implementation of VIT, the identification of patients at high risk for severe sting reactions and the adjustment of treatment protocols and times.

The detailed mechanisms of tolerance induction during VIT are not completely understood. Nevertheless, several immunological changes, which are associated with the success of therapy, are well described. Venom-specific regulatory T cells (Treg) and Th1 cells are thought to be induced during VIT and are able to suppress pro-allergic Th2 cells. Further, the Th2 suppression leads to reduction of the levels of cytokines such as IL-4, IL-5, IL-9, and IL-13 resulting in a desensitization of mast cells and basophils (191). Moreover, an induction of specific IgG4 antibodies might be of relevance as blocking antibodies are supposed to have a protective anti-inflammatory role (192). Additionally, the induction of B cells with a regulatory phenotype (Breg) was shown to be an important event during VIT (193). Bregs are able to suppress venom-specific T cell proliferation (194) and to induce Tregs (195, 196), thus, boosting the shift towards a tolerogenic phenotype.

VIT is performed by subcutaneous injections of whole venom extracts. The suggested maintenance dose of 100 µg can be reached using different protocols. In conventional protocols, maintenance dose is reached in several weeks to month, whereas in rush and ultra-rush protocols that use several injections per day on consecutive days, maintenance dose is reached within a few days or hours, respectively. In cluster protocols, patients receive several injections per day in intervals of 1–2 weeks. Intervals between maintenance injections can be gradually increased from 4 weeks (first year) to 6 (second year) and 8 weeks (in case of a 5-year treatment from year 3–5) without loss of clinical protection (21, 197).

Several studies showed that in most patients clinical protection is achieved as soon as the maintenance dose is reached (190, 198). Most of the patients who are still reacting to a sting while receiving the conventional maintenance dose of 100 µg will be protected by increased venom dosages during VIT (74, 199). VIT is reportedly effective in preventing future SRs in 77%–84% of patients treated with HBV, 91%–96% of patients receiving vespid venom (200, 201), and 97%–98% of patients treated with ant venom (202, 203). The reasons for the lower efficacy of VIT with HBV are still unclear. Potential explanations might be the much larger and consistent venom amount delivered by a honeybee sting (204) or the broad sensitization profiles of HBV-allergic patient with different major HBV allergens (103), including those that might be underrepresented in certain therapeutic venom preparations (73, 166).

VIT should be performed for 3–5 years, whereby most experts recommend 5 years (120). Of note, stopping VIT after 3 years might only be feasible for patients with mild to moderate reactions and should not be done when sting challenge during therapy cannot be performed (205, 206). VIT with a minimum duration of 5 years is superior for long-term effectiveness and protects the majority of patients (207, 208). A recent study on the outcome of re-stings on a long follow-up period after VIT discontinuation (up to 26 years) showed a very low risk of relapse (3.4%) in patients treated on average for about 10 years (209).

According to some studies, risk factors that are associated with a loss of protection after discontinuation of VIT include very severe initial SRs, systemic adverse events during VIT (injection or sting), treatment of less than 5 years, elevated basal serum tryptase and/or mastocytosis, HBV allergy, cardiovascular disease and others (21, 197). However, all patients continue to have a 10% chance of having a reaction to a future sting (210) and the only way to keep the risk down to 2% is to remain on maintenance VIT (205). Lifelong therapy should be particularly considered in high-risk patients such as those suffering from mastocytosis as well as in patients at high risk for future stings such as beekeepers. A recent study on the outcome of re-stings on a long follow-up period after VIT discontinuation (up to 26 years) showed a very low risk of relapse (3.4%) in patients treated on average for about 10 years (209).

## Conclusions

In the first placebo-controlled trial in 1978, allergen-specific immunotherapy with insect venoms has proven to be superior over therapy with whole body extracts of the insects (211) and since then demonstrated to be a highly effective curative treatment of venom allergy. Nevertheless, the growing knowledge of different disease manifestations of HVA and of disease-influencing comorbidities has increasingly improved adequate diagnostics and patient management. For instance, the availability of CRD has facilitated the differentiation of primary allergy and cross-reactivity and, thus, therapeutic decisions in multiple-sensitized patients. Moreover, biomarkers such as the c-kit D816V mutation or elevated baseline tryptase levels that allow to identify patients at risk for very severe sting reactions were identified and allow a personalized patient management. Nevertheless, there is a need for additional biomarkers which reliably allow therapy monitoring, the identification of potential VIT non-responders and patients at risk for severe side effects as well as to monitor immunological tolerance after discontinuation of VIT. There is some evidence that the analysis of patients’ sensitization profile might help to predict the outcome of VIT in the future, to better adjust treatment strategies and to select the most suitable venom preparation in a personalized manner (73, 167).

To further classify endotypes and phenotypes in HVA might be a promising approach to better understand the disease, to strengthen personalized treatment strategies and, thus, precision medicine in HVA. Moreover, detailed molecular analyzes of the immunological processes occurring during VIT might contribute to a deeper understanding of immune tolerance to allergens. This in turn can support the development of novel immunomodulatory strategies that might enhance tolerance induction as well as the identification of new biomarkers that indicate therapeutic success in an early state of treatment.

## Author Contributions

All authors contributed to the article and approved the submitted version.

## Funding

This work was supported by the Helmholtz Association, Future Topic “Immunology and Inflammation” (ZT-0027) to SB, by the Personalized Medicine Consortium (PMC) of Luxembourg (SYS-T-ACT) to MO and the FNR PRIDE Doctoral Training Unit program (PRIDE/11012546/NEXTIMMUNE) to MO. The publication of this article was supported by Bencard Allergy GmbH, Munich, Germany. The funder was not involved in the study design, collection, analysis, interpretation of data, the writing of this article or the decision to submit it for publication.

## Conflict of Interest

SB reports nonfinancial support from ALK-Abelló, grants, personal fees and nonfinancial support from Bencard Allergie GmbH, personal fees from Teomed AG, grants from Leti Pharma, grants and personal fees from Thermo Fisher Scientific, grants from Allergy Therapeutics, outside the submitted work. MO reports personal fees from Thermo Fisher Scientific, Siemens Healthcare Diagnostics, Hitachi Chemical Diagnostics and Hycor, outside the submitted work; and is Scientific co-founder of the academic biotech spin-offs PLS-Design GmbH, Hamburg, Germany and Tolerogenics SARL; Luxembourg. MB has received speaker’s honorarium and consultancy fees from ALK-Abelló, outside the submitted work.

The remaining author declares that the research was conducted in the absence of any commercial or financial relationships that could be construed as a potential conflict of interest.
